# The Differential Responses of Coastal Diatoms to Ocean Acidification and Warming: A Comparison Between *Thalassiosira* sp. and *Nitzschia closterium f.minutissima*

**DOI:** 10.3389/fmicb.2022.851149

**Published:** 2022-06-21

**Authors:** Ting Cai, Yuanyuan Feng, Yanan Wang, Tongtong Li, Jiancai Wang, Wei Li, Weihua Zhou

**Affiliations:** ^1^School of Oceanography, Shanghai Jiao Tong University, Shanghai, China; ^2^Key Laboratory of Marine Ecosystem Dynamics, Ministry of Natural Resources, Hangzhou, China; ^3^Shanghai Frontiers Science Center of Polar Science, Shanghai, China; ^4^College of Marine and Environmental Sciences, Tianjin University of Science and Technology, Tianjin, China; ^5^CAS Key Laboratory of Tropical Marine Bio-Resources and Ecology, South China Sea Institute of Oceanology, Chinese Academy of Sciences, Guangzhou, China; ^6^Key Laboratory of Tropical Marine Biotechnology of Hainan Province, Sanya Institute of Oceanology, South China Sea Institute of Oceanology, Chinese Academy of Sciences, Sanya, China; ^7^Sanya National Marine Ecosystem Research Station and Tropical Marine Biological Research Station in Hainan, Chinese Academy of Sciences, Sanya, China

**Keywords:** ocean acidification, warming, diatoms, sinking rate, biogeochemistry

## Abstract

Marine diatoms are one of the marine phytoplankton functional groups, with high species diversity, playing important roles in the marine food web and carbon sequestration. In order to evaluate the species-specific responses of coastal diatoms to the combined effects of future ocean acidification (OA) and warming on the coastal diatoms, we conducted a semi-continuous incubation on the large centric diatom *Thalassiosira* sp. (~30 μm) and small pennate diatom *Nitzschia closterium f.minutissima* (~15 μm). A full factorial combination of two temperature levels (15 and 20°C) and pCO_2_ (400 and 1,000 ppm) was examined. The results suggest that changes in temperature played a more important role in regulating the physiology of *Thalassiosira* sp. and *N. closterium f.minutissima* than CO_2_. For *Thalassiosira* sp., elevated temperature significantly reduced the cellular particulate organic carbon (POC), particulate organic nitrogen (PON), particulate organic phosphate (POP), biogenic silica (BSi), chlorophyll a (Chl *a*), and protein contents, and the C:N ratio. CO_2_ only had significant effects on the growth rate and the protein content. However, for the smaller pennate diatom *N. closterium f.minutissima*, the growth rate, POC production rate, and the C:P ratio significantly increased with an elevated temperature, whereas the cellular POP and BSi contents significantly decreased. CO_2_ had significant effects on the POC production rate, cellular BSi, POC, and PON contents, the C:P, Si:C, N:P, and Si:P ratios, and sinking rate. The interaction between OA and warming showed mostly antagonistic effects on the physiology of both species. Overall, by comparison between the two species, CO_2_ played a more significant role in regulating the growth rate and sinking rate of the large centric diatom *Thalassiosira* sp., whereas had more significant effects on the elemental compositions of the smaller pennate diatom *N. closterium f.minutissima*. These results suggest differential sensitivities of different diatom species with different sizes and morphology to the changes in CO_2_/temperature regimes and their interactions.

## Introduction

The atmospheric CO_2_ concentration has increased by about one-third over pre-industrial levels, with a continuing increase of around 0.4% per year. By the end of this century, atmospheric CO_2_ levels are expected to increase to 800–1,000 ppm (IPCC, 2021). On the one hand, the carbonate buffer system in the surface ocean will be profoundly influenced when oceans have absorbed approximately one-third of all anthropogenic CO_2_ released into the atmosphere (Wolf-Gladrow et al., 1999). With rising CO_2_ level in the atmosphere, the concentration of hydrogen ion in seawater will increase (Orr, 2005), whereas pH will be reduced, causing ocean acidification (OA) (Caldeira and Wickett, 2003). On the other hand, atmospheric CO_2_ plays an important role in global climate regulation by changing the earth's radiation budget, temperature, meteorology, and hydrology. Over the past 100 years, global average temperature has risen by 0.6–0.2°C (IPCC, 2021), mainly because of the release of CO_2_ and other greenhouse gases into the atmosphere. Models have predicted that sea surface temperature will be elevated by at least a further 1–4°C in some areas of the ocean by the end of this century (Bopp et al., 2001).

These concurrent trends of OA and warming will directly affect marine organisms and alter the structure and function of marine ecosystems (Gattuso et al., 2015). The trend of CO_2_ enrichment will have a large effect on the photosynthesis, calcification, and elemental composition of marine phytoplankton (Tortell et al., 2002; Feng et al., 2009; Schulz et al., 2013; Neale et al., 2014). The concentration of carbon dioxide in seawater may affect phytoplankton physiology and ecology and their role in marine biogeochemical cycles, thus having profound impact on biological carbon pump (Riebesell et al., 2009). Elevated temperature will lead to accelerated metabolic activity, and the growth rate of phytoplankton is generally positively correlated with temperature within a suitable range (Talling, 1955; Eppley, 1972). Due to the different sensitivities to warming among various phytoplankton functional groups, the phytoplankton biogeographical distribution and phytoplankton community composition have been recorded to change in the world ocean (Winter et al., 2014; Anderson et al., 2021).

Diatoms are one of the marine phytoplankton functional groups, with the unique feature of producing silica cell walls (frustules) (Martin-Jézéquel et al., 2000), responsible for ~40% of total oceanic primary productivity (Nelson et al., 1995). In addition, large species and cell size diversities (Von Dassow et al., 2008; Bowler et al., 2010) have been observed within diatoms. Diatoms possess a highly efficient CO_2_-concentrating mechanism (CCM) that allows the cells to maintain good photosynthetic performance under conditions of low CO_2_ concentrations in the marine environment (Giordano et al., 2005; Gao and Campbell, 2014; Matsuda et al., 2017). Therefore, an elevated CO_2_ concentration tends to affect the photosynthesis of diatoms to a less extend than other phytoplankton groups. In the meanwhile, cell size often affects the physiological processes of diatoms, including nutrients absorption and utilization efficiencies (Finkel et al., 2009; Li and Campbell, 2017), photosynthetic capacity (Key et al., 2010; Li and Gao, 2013), and growth response (Marañón et al., 2013; Wu et al., 2014). Previous studies also showed that the physiological responses to CO_2_ may differ among different diatom species (Gao and Campbell, 2014), with small pennate diatom species being favored more than large centric diatoms (Tortell et al., 2008). In addition, it has been suggested that temperature may regulate the physical responses of diatoms to OA (Li et al., 2021) and the natural diatom communities (Feng et al., 2021). There has been a large body of studies on the responses of marine diatoms to OA and/or temperature changes published during the past decades (see the summary in Table 1). However, studies on the species-specific responses of diatoms to the interaction between OA and warming are still scarce. For a better projection of the response natural diatom communities to the future greenhouse scenario, the potential interactive effects of OA and warming on different diatom species in terms of the important traits of cell sizes and morphology require further investigation.

**Table 1:** Response of diatoms to rising pCO_2_ and elevated temperature summarized from published studies, considering incubation conditions in terms of growth rate (μ), cellular particulate organic carbon (POC) content, cellular particulate organic nitrogen (PON) content, cellular particulate organic phosphate (POP) content, cellular biogenic silica (BSi) content, the POC-to-PON ratio (C:N), the POC-to-POP ratio (C:P), the PON-to-POP ratio (N:P), the BSi-to-POC ratio (Si:C), the lipid content, the protein content, and the carbohydrate content.

**Class**	**Diatom Species**	**Factor (Condition)**	**μ**	**Cell Size**	**Cellular POC**	**Cellular PON**	**Cellular POP**	**Cellular BSi**	**C:N**	**C:P**	**N:P**	**Si:C**	**Lipid**	**Protein**	**Carbohydrate**	**Reference**
Centric	*Thalassiosira pseudonana*	150–900 ppm	↔	41 (0.2) μm^3^	↓	↓	↓		↑	↑	↑					(Reinfelder, 2012)
	*Thalassiosira weissflogii*	150–900 ppm	↔	626 (6) μm^3^	↑	↓	↓		↑	↑	↑					(Reinfelder, 2012)
	*Thalassiosira weissflogii*	410 ppm 1000 ppm	↑			↓	↓	↓	↑							(Qu et al., 2018)
	^*^Chaetoceros gracilis	Air bubbling 3% CO_2_	↑													(Nagao et al., 2020)
	^*^Skeletonema dohrnii	400 ppm; 1,000 ppm	↑										↑	↑	↑	(Thangaraj and Sun, 2020)
	^*^Thalassiosira sp.	400 ppm; 1,000 ppm	↔	28 μm	↓	↔	↓	↓	↓	↔	↑	↓		↓	↑	This study
	*Coscinodiscus* sp.	16°C; 20°C	↑		↔	↔	↓	↓			↑	↓				(Qu et al., 2018)
	*Stephanodiscus minutulus*	5°C; 10°C; 15°C; 20°C	↑					↑								(Shatwell et al., 2013)
	*Skeletonema costatum*	8°C; 13°C; 18°C; 23°C	↑↓					↑				↑				(Paasche, 1980)
	*Thalassiosira pseudonana*	8°C; 13°C; 18°C; 23°C	↑					↑↓				↑				(Paasche, 1980)
	*Chaetoceros affinis*	8°C; 13°C; 18°C; 23°C	↑↓					↓				↓				(Paasche, 1980)
	*Rhizosolenia fragilissima*	8°C; 13°C; 18°C; 23°C	↑					↓				↑↓				(Paasche, 1980)
	*Cerataulina pelagica*	8°C; 13°C; 18°C; 23°C	↑					↔				↑				(Paasche, 1980)
	*Chaetoceros wighamii*	3°C; 7°C; 11°C;	↑						↓	↓	↔					(Spilling et al., 2015)
	^*^Chaetoceros gracilis	25°C; 30°C	↑													(Nagao et al., 2020)
	^*^Skeletonema dohrnii	21°C; 25°C	↑										↑	↑	↑	(Thangaraj and Sun, 2020)
	^*^Thalassiosira sp.	20°C 25°C	↑	28 μm	↓	↓	↓	↓	↔	↑	↓	↔		↓	↔	This study
Pennate	*Phaeodactylum tricornutum*,	150–900 ppm	↔	65	↔	↔	↓		↑	↑	↑					(Reinfelder, 2012)
	^*^Amphora coffeae	400 ppm; 750 ppm	↑		↑	↔	↔		↑	↑	↔					(Tew et al., 2014)
	^*^Nitzschia ovalis	400 ppm; 750 ppm	↔		↑	↑	↔		↓	↑	↑					(Tew et al., 2014)
	^*^Navicula phyllepta	380 ppm; 960 ppm	↑													(Sabu et al., 2017)
	^*^Nitzschia lecointei	400 ppm; 1,000 ppm	↑		↓	↓			↑				↑	↑	↑	(Torstensson et al., 2019)
	^*^N. closterium f.minutissima	400 ppm; 1,000 ppm	↓	15 μm	↓	↑	↓	↓	↑	↑	↑	↓		↓	↑	This study
	*Nitzschia acicularis*	5°C; 10°C	↑					↑								(Shatwell et al., 2013)
	*Nitzschia cf. neglecta*	2–10°C	↑													(Yan et al., 2019)
	*Amphora* sp. *MUR258*	24°C; 35°C	↑										↓			(Indrayani et al., 2020)
	*Navicula directa*	0.5°C; 4.5°C	↑													(Torstensson et al., 2012)
	^*^Amphora coffeae	28°C; 31°C	↓		↔	↑	↔		↓	↑	↑					(Tew et al., 2014)
	^*^Nitzschia ovalis	28°C; 31°C	↔		↑	↑	↔		↓	↔	↔					(Tew et al., 2014)
	^*^Navicula phyllepta	20°C; 30°C	↑										↑			(Sabu et al., 2017)
	^*^Nitzschia lecointei	−1.8 to 3°C	↑						↓				↓	↑	↓	(Torstensson et al., 2019)
	^*^N. closterium f.minutissima	20°C; 25°C	↑	15 μm	↑	↑	↓	↓	↑	↑	↑	↓		↓	↔	This study

“*↑”Represents increase, “↓” represents decrease, and “↔” represents no significant change*.

* *Represents the combined effects of ocean warming and acidification on the centric and pennate diatom's exploitation*.

The aim of this study was to examine the individual and interactive effects and the potential species-specific responses of OA and warming on two coastal diatom species: a large-celled centric *Thalassiosira* sp. (~30 μm) and a small-celled pennate diatom *Nitzschia closterium f.minutissima* (~15 μm). The study was carried out in full factorial combination with two levels of CO_2_ (400 and 1,000 ppm) and temperatures (15 and 20°C). The experimental conditions of a CO_2_ level of 1,000 ppm and 20°C (control+5°C) are based on the high-emission scenario [representative concentration pathway (RCP) 8.5] projected for the end of this century (IPCC, 2021), which will represent a global mean warming ranging between 2.6 and 4.8°C and an atmospheric CO_2_ level exceeding 900 ppm (Riebesell and Tortell, 2011).

## Materials and Methods

### Stock Cultures and Laboratory Incubation Experiment

The marine diatom *Thalassiosira* sp. and *N. closterium f.minutissima* were isolated from the surface water (depth of 3–12 m, salinity of 34.78) at 118°58.055′E, 38°39.111′N, China, by boarding R/V “BeiDou” in August 2019. The sea surface temperature at the sampling site for the isolation was 15°C. Therefore, the stock cultures were maintained in the laboratory at 15°C and irradiance of ~50 μmol photon m^−2^ s^−1^ under a light: dark cycle of 12 h:12 h. The medium used for maintaining the stock culture was seawater obtained from the Yellow Sea, China, filtered using 0.2-μm pore size filtration cartridges (Whatman, USA) and supplemented with nutrient according to the f/2 recipe (Guillard and Ryther, 1962).

For the semi-continuous incubation experiments, the cells of *Thalassiosira* sp. and *N. closterium f.minutissima* were grown in acid-cleaned 500-ml Nalgene bottles under the interactive effect of different temperatures (15 and 20°C) and pCO_2_ (400 and 1,000 ppm) conditions for >20 generations. Cultures were preadapted to the experimental conditions for 2–3 days and maintained dilute by starting the experiment with low cell concentrations (~1 × 10^4^ cells mL^−1^). For the manipulation experiments, final concentrations of 100 μM nitrate, 6 μM phosphate, and 100 μM silicate were added in the medium. Trace metal and vitamin were added according to the f/2 recipe (Guillard and Ryther, 1962). Both species were cultured under an irradiance of ~160 μmol photon m^−2^ s^−1^ and a light/dark cycle of 12 h/12 h. The CO_2_ level of 400 ppm was reached by pre-aerating the medium with the ambient outdoor air, whereas the CO_2_ level of 1,000 ppm was achieved by adding medium with saturated CO_2_ in a closed system. The level of 1,000 ppm pCO_2_ was chosen according to the “Guide to the Best Practices for Ocean Acidification Research and Data Reporting” to represent the future condition of the end of this century (Riebesell and Tortell, 2011). The concentrations of dissolved inorganic carbon (DIC) at different pCO_2_ levels were estimated using CO2SYS (Dickson and Millero, 1987). To minimize effects of photosynthesis and respiration on the carbonate system, the cell abundances were kept <6 × 10^4^ cells mL^−1^ with cells still growing in the exponential phase. As such, <4% of the DIC in the medium had been taken up by the cells, causing a shift in pH not more than 0.15 units (Supplementary Table S1).

The pH, the total alkalinity (TA), and the *in vivo* chlorophyll a (Chl *a*) fluorescence were monitored prior to the daily dilution within 2 h of the start of the light period. The *Thalassiosira* sp. and *N. closterium f.minutissima* were grown under different treatments for about 25 days. Sampling for all analyses was carried out after the cultures had maintained steady-state growth rates for >7 generations (Feng et al., 2008) within 2 h of the start of the light period on the final sampling day.

### Physiological and Biogeochemical Analyses

#### Seawater Bicarbonate Chemistry

The pH was measured using a pH meter (Mettler Toledo DL15 Titrator, Sweden), calibrated with National Bureau of Standards (NBS) buffer solutions each time before using. TA was measured using potentiometric titration following the method of Dickson and Millero (1987). The accuracy of the method, as determined by the analysis of Certified Reference Material (Andrew Dickson, Scripps Institution of Oceanography) was ±2 mmol kg^−1^. The seawater carbonate chemistry was calculated using the program CO2SYS version 1.05, using the constants in Mehrbach et al. (1939), refitted by Dickson and Millero (1987).

#### Cell Counts and Growth Rates

Sample for cell counts were preserved in the dark at 4°C by adding Lugol's solution, using a Zeiss Microscope (Axiostar Plus, Germany). During the semi-continuous incubation, real-time biomass was estimated with *in vivo* fluorescence before and after dilution. The *in vivo* Chl *a* fluorescence was measured using a fluorometer (Trilogy, Turner Designs, USA), as an indicator of Chl *a* biomass and cell growth (Gilbert et al., 2000).

Growth rates (*m*) were calculated as described previously (Brading et al., 2011) using *in vivo* Chl *a* fluorescence daily as:


(1) $$\begin{array}{r} \mu =LN\left(\frac{N_{T2}-N_{T1}}{T2-T1}\right), \end{array}$$

where *N*_T1_ and *N*_T2_ are the *in vivo* Chl *a* fluorescence values at time points of T1 and T2.

#### Biomolecules and Particulate Organic Matters

In total, 20 ml of sample for Chl *a* analysis was filtered through GF/F filters (Whatman^®^). After adding 5 ml of 90% acetone, Chl *a* was extracted in the freezer at −20°C and measured using the acidification method using a fluorometer (Trilogy, Turner Designs) after 24 h (Welschmeyer, 1994).

The samples for particulate organic carbon (POC) and particulate organic nitrogen (PON) analyses were collected by vacuum filtration onto precombusted (450°C for 3 h) GF/F filters, oven-dried (60°C), and stored in a desiccator. The samples were analyzed on a PerkinElmer Series II CHNS/O Analyzer 2400 using acetanilide as the calibration standard (Hilton et al., 1986). The POC production rate was calculated by multiplying the cellular POC content by growth rate (μ).

For biogenic silica (BSi) analyses, samples were collected onto 0.6 μm polycarbonate membrane filters (Millipore, USA), dried at 60°C, and then measured with a spectrophotometric method (Nelson et al., 1995).

Samples for particulate organic phosphate (POP) measurements were filtered onto precombusted (450°C for 3 h) GF/F filters (Whatman^®^), rinsed with 0.017 M Na_2_SO_4_ solution, and then dried at 60°C until analysis following the molybdate colorimetric method (Solórzano and Sharp, 1980).

Protein samples were collected on 0.6-μm polycarbonate filters by gentle filtration (0.2 bar) of the cells and subsequently stored −80°C until further analysis. Samples were extracted in 1 ml distilled water and pipetted 4-ml coomassie brilliant blue G-250 (CBBG) (dissolve 100 mg CBBG in 50 ml 90% methanol, add 100 ml 85% phosphoric acid, dilute to 1 L with distilled water). The mixtures were incubated at room temperature for 10 min. Absorbance spectra were scanned at 595 nm wavelength, and protein concentration was calculated using bovine serum albumin (BSA) as standard (Cheng et al., 2018).

The samples for analyzing carbohydrates were filtered into 0.6 μm polycarbonate and subsequently stored until further analysis. Samples were extracted in distilled water and heated at 60°C for 2 h. Carbohydrates were measured in the supernatant using the phenol-sulfuric acid (PSA) method (Dubois et al., 1956). In brief, 2 ml sample was mixed with 50 μl 80% phenol and 5 ml concentrated H_2_SO_4_ and left to react for 30 min. The absorbance of the sample was read at 485 nm and carbohydrate concentration was calculated using glucose as standard.

#### Sinking Rate

The sinking rate was measured using the SETCOL method (Bienfang, 1981). For the analysis, a plexiglass column with height of 33 cm and volume of 420 ml was filled completely with the original sample. A tight-fitting cap was placed on the column to remove free surface area, thus inhibiting water motion. Then the plexiglass column was allowed to settle undisturbed for 3 h. The SETCOL apparatus was then kept in the dark with neutral density screen covering the columns during the incubation. Additionally, the original sample has been kept outside of the column under identical conditions. The purpose of this measurement was to account for any biomass changes during the trial due to processes other than sinking. Upon completion of the trial, the upper, middle, and bottom regions were slowly decanted by gradually opening a valve to allow flow (< 50 ml min^−1^) through a side port in order. The volume was then precisely measured, and the contents were filtered onto GF/F filters (Whatman^®^) and then kept in the dark at 4°C for Chl *a* measurement. The sinking rates were calculated using the following equation:


(2) $$\begin{array}{r} S=\frac{C_{b}\times V_{b}}{C_{u}\times V_{u}+C_{m}\times V_{m}+C_{b}\times V_{b}}\times \frac{l}{t} \end{array}$$

where S is the sinking rate; C_u_, C_m_, and C_b_ are the Chl *a* concentrations in the upper, middle, and bottom compartments of the sinking column, respectively; *V*_u_, *V*_m_, and *V*_b_ are the volumes in the upper, middle, and bottom compartments, respectively; *l* is the length of the column; and *t* is the total sinking time.

The average cell size was measured using a laser particle size analyzer (LS 13320, Beckman, USA). Triplicate samples of ~50 ml cultures were measured for cultures taken from each incubation bottle.

### Data Analyses

The interactive effects of CO_2_ and temperature on the physiological rates of *Thalassiosira* sp. and *N. closterium f.minutissima* were determined with two-way analysis of variance (two-way ANOVA) using GraphPadPrism 7.0 software (GraphPad Software, Inc., San Diego, CA, United States). Differences between treatments were considered significant at level of *p* < 0.05. The pairwise tests between treatments were conducted using Tukey's multiple comparison *post-hoc* analysis.

Two-way interactions between temperature and pCO_2_ were determined by quantitative comparisons between the observed effects and the model predicted effects of two drivers. The observed effect was calculated as the percentage of change between the combined treatment and control treatment. A model was used for the predictions of the multiplicative effect (ME):


(3) $$\begin{array}{r} ME_{\text{A}+\text{T}}=\left(1+OE_{\text{A}}\right)\times \left(1+OE_{\text{T}}\right)-1 \end{array}$$

where OE_A_ and OE_T_ denote the individual observed effect of increased pCO_2_ and temperature on the measured physiological parameter, respectively, calculated as the percentage of changes relative to control treatment. The interactive effects of CO_2_ and temperature were defined according to Feng et al. (2021). Synergistic interactive effect: the observed effect > the calculated multiplicative effect; antagonistic interactive effect: the observed effect < the calculated multiplicative effect (Folt et al., 1999; Boyd and Hutchins, 2012; Boyd et al., 2018). Both synergistic and antagonistic interactive effects can be positive (increase) or negative (decrease).

## Results

### Growth and POC Production Rates

**Figure 1:**
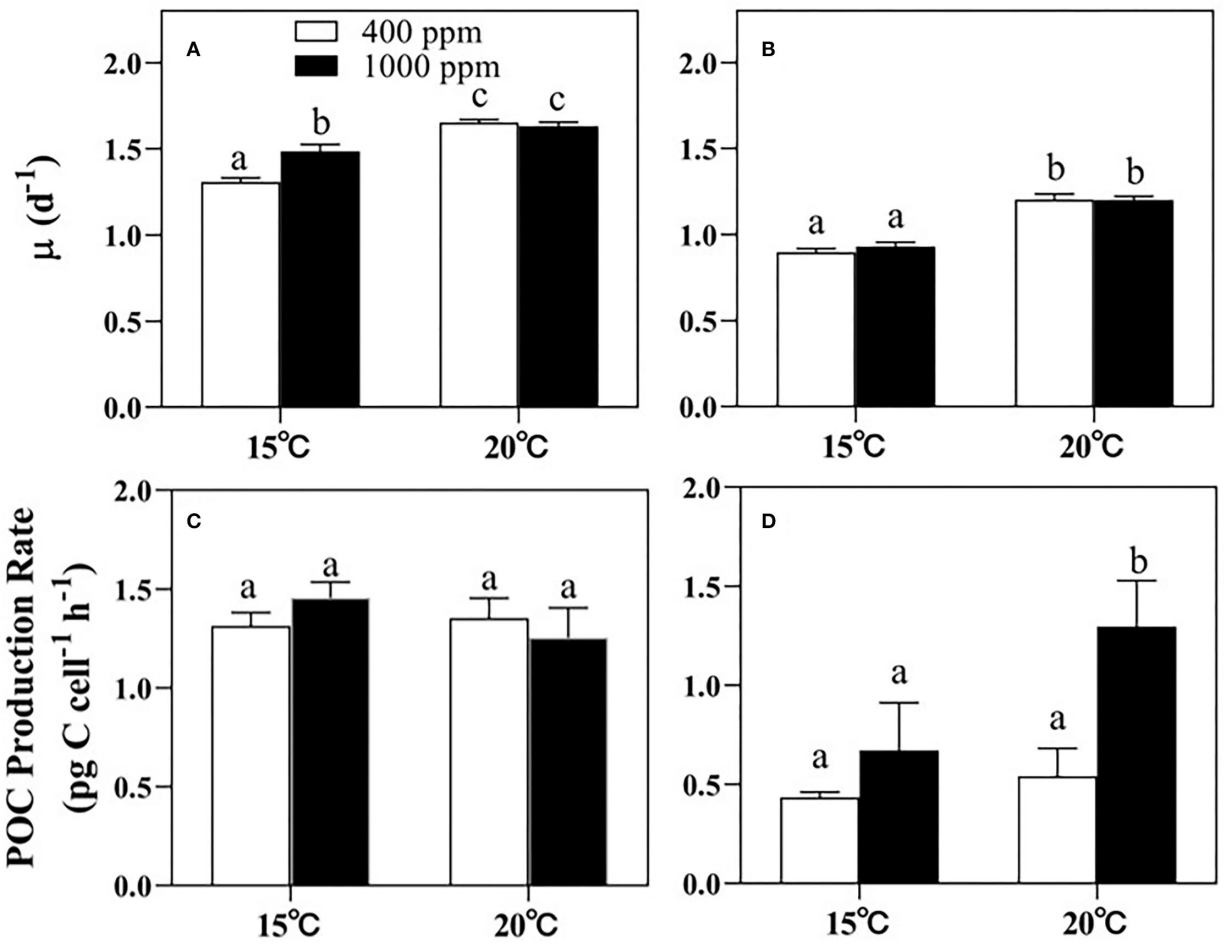
The growth rates and the POC production rates of *Thalassiosira* sp. **(A,C)** and *N. closterium f.minutissima* **(B,D)** under different pCO_2_ and temperature conditions. The error bars represent standard deviations of triplicate samples. The letters above the bars denote the statistically significant differences. POC, particulate organic carbon.

Warming played a more significant role than changes in pCO_2_ in regulating the growth of both *Thalassiosira* sp. and *N. closterium f.minutissima* (*p* < 0.05, Figure 1). For *Thalassiosira* sp., the growth rate was increased by 26.38% and 9.88% by 5°C of warming under 400 and 1,000 ppm pCO_2_, respectively (Figure 1A), whereas the growth rate of *N. closterium f.minutissima* was increased by 34.09% and 28.99% under the two pCO_2_ levels (Figure 1B). Rising pCO_2_ only promoted the growth of *Thalassiosira* sp. at 15°C (Figure 1A). There was no significant effects of changes in pCO_2_ on the growth of *N. closterium f.minutissima*.

However, the POC production rate of *Thalassiosira* sp. was not affected by either warming or increased pCO_2_ (Figure 1C). In contrast, for *N. closterium f.minutissima*, the POC production rate was increased by 93.17% under 1,000 ppm pCO_2_ by warming, respectively (*p* < 0.05, Figure 1D). In addition, increased pCO_2_ also significantly increased the POC production rate by 54.52 and 139.2%, respectively, under the two temperature conditions (*p* < 0.05, Figure 1D).

### Elemental Stoichiometry

**Figure 2:**
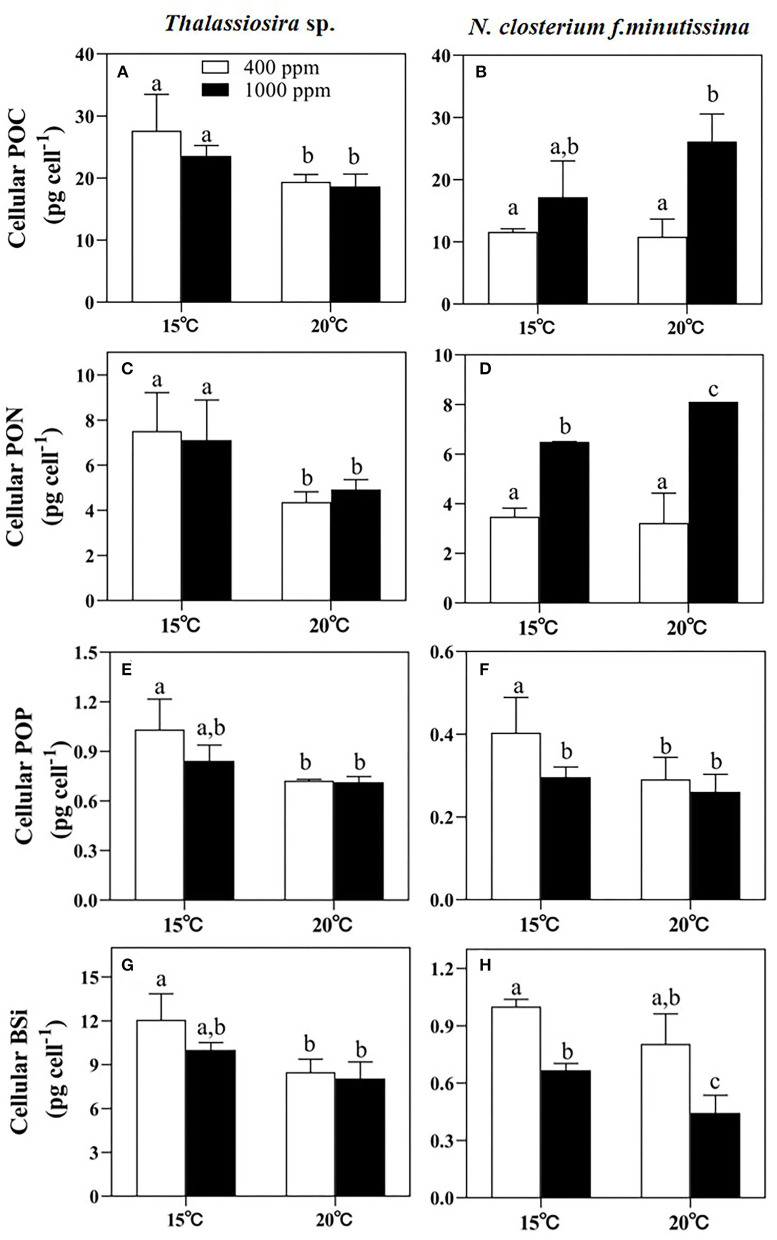
The cellular POC, PON, POP, and BSi contents of *Thalassiosira* sp. **(A,C,E,G)** and *N. closterium f.minutissima* **(B,D,F,H)** under different pCO_2_ and temperature interactions. The error bars represent standard deviations of triplicate samples. The letters above the bars denote the statistically significant differences. POC, particulate organic carbon; PON, particulate organic nitrogen; POP, particulate organic phosphate; BSi, biogenic silica.

Significant temperature effects on the cellular POC, PON, POP, and BSi contents of *Thalassiosira* sp. were observed (*p* < 0.05, **Table 3**). Warming significantly decreased the cellular POC, PON, POP, and BSi contents under both pCO_2_ conditions (Figure 2). Difference from what observed for *Thalassiosira* sp., the cellular POC and PON contents of *N. closterium f.minutissima* were both increased under higher pCO_2_ (*p* < 0.05, Figures 2B,D) when temperature was the same. Rising pCO_2_ significantly increased the cellular POC content of *N. closterium f.minutissima* by 141.6% at 20°C and significantly increased the cellular PON content by 86.59% and 151.5% at 15 and 20°C, respectively. Increased pCO_2_ only significantly decreased the cellular POP and BSi contents of *Thalassiosira* sp. at 15°C. However, this trend was not observed at 20°C (Figures 2E,G). Similarly, warming significantly decreased the cellular BSi content of *N. closterium f.minutissima* under both 400 and 1,000 ppm conditions, but only significantly caused a decrease in the cellular POP content at 400 ppm pCO_2_ (Figures 2F,H).

The ratio of the cellular POC to PON (C:N) and the ratio of cellular POC to POP (C:P) of *Thalassiosira* sp. were significantly affected by warming (*p* < 0.05, **Table 3**). The C:N increased, whereas the C:P decreased with an elevated temperature at both 400 and 1,000 ppm pCO_2_. The PON-to-POP ratio (N:P) decreased with an elevated temperature (*p* < 0.05, **Table 3**), but there was no significant effect of CO_2_. For *N. closterium f.minutissima*, the C:P, Si:C, and N:P values were all significantly increased under 1,000 ppm pCO_2_ compared to those at 400 ppm (*p* < 0.05) when temperature was the same, contrary to the trend of Si:P. Similarly, the C:P and N:P ratios increased, whereas the Si:C ratio decreased with an elevated temperature between different pCO_2_ treatments (Table 2).

**Table 2:** The cell size, POC-to-PON ratio (C:N), the POC-to-POP ratio (C:P), the BSi-to-POC ratio (Si:C), the PON-to-POP ratio (N:P) and the BSi-to-POP ratio (Si:P) (molar ratio) of *Thalassiosira* sp. and *N. closterium f.minutissima* under different pCO_2_ and temperature conditions (the errors represent standard deviations of triplicate samples; the letters (a, b, and c) denote the statistically significant differences).

**Treatment**	***Thalassiosira*** **sp**.						***N. closterium f.minutissima***					
	**Cell size (μm)**	**C:N**	**C:P**	**Si:C**	**N:P**	**Si:P**	**Cell size (μm)**	**C:N**	**C:P**	**Si:C**	**N:P**	**Si:P**
15 °C + 400 ppm	28.39 (0.89)^a^	4.30 (0.09)^a^	68.96 (4.91)^a^	0.20 (0.003)^a^	16.07 (1.48)^a^	13.01 (0.83)^a^	15.99 (1.16)^a^	3.92 (0.37)^a^	75.88 (12.08)^a^	0.04 (0.001)^a^	19.66 (4.69)^a^	2.82 (0.53)^a^
15°C + 1,000 ppm	27.65 (1.85)^a^	3.97 (0.70)^a^	72.32 (3.07)^a^	0.18 (0.004)^a^	18.49 (2.56)^a^	13.20 (0.81)^a^	15.75 (0.42)^a^	3.84 (0.38)^a^	177.8 (36.20)^b^	0.02 (0.008)^b^	33.65 (15.21)^b^	2.51 (0.33)^a^
20°C + 400 ppm	28.39 (1.73)^a^	5.21 (0.25)^a^	69.42 (4.80)^a^	0.19 (0.009)^a^	13.38 (1.55)^a, b^	13.03 (1.49)^a^	15.46 (0.06)^a^	4.07 (0.73)^a^	95.43 (10.77)^a^	0.03 (0.003)^a^	23.93 (4.86)^a^	3.06 (0.08)^a^
20°C + 1,000 ppm	28.45 (2.33)^a^	4.44 (0.64)^a^	67.34 (4.76)^a^	0.18 (0.009)^a^	15.27 (1.17)^a^	12.47 (1.21)^a^	14.79 (0.68)^a^	N/A	236.9 (23.49)^b^	0.007 (0.001)^c^	N/A	1.91 (0.46)^a, b^

*N/A represents missing value due to loss of samples*.

### Biomolecules

**Figure 3:**
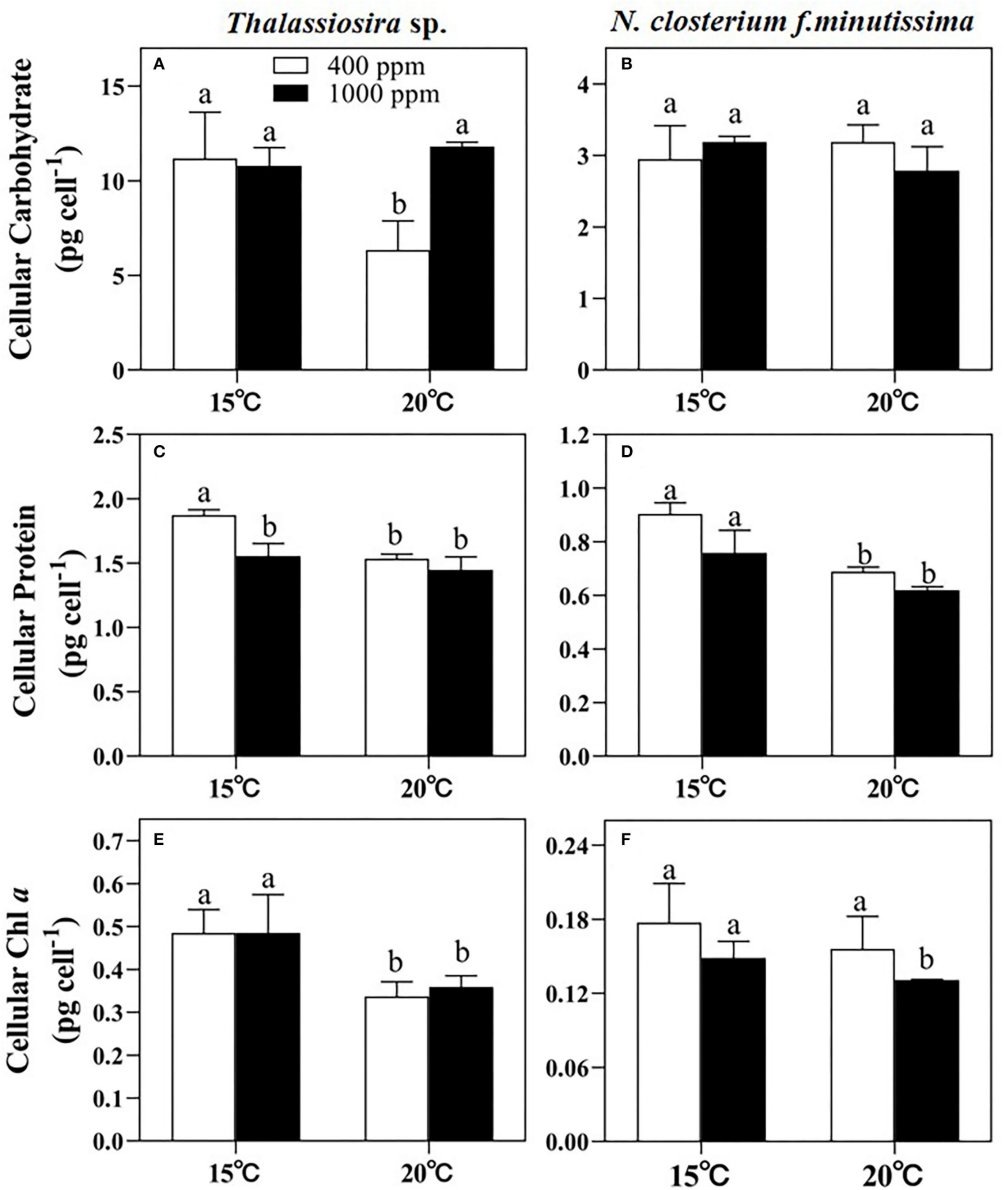
The cellular carbohydrate, protein, and Chl *a* contents of *Thalassiosira* sp. **(A,C,E)** and *N. closterium f.minutissima* **(B,D,F)** under different pCO_2_ and temperature conditions. The error bars represent standard deviations of triplicate samples. The letters above the bars denote the statistically significant differences.

Significant temperature effects on the cellular carbohydrate content of *Thalassiosira* sp. were observed (*p* < 0.05, Figure 3A and Table 3). However, for *N. closterium f.minutissima*, the cellular carbohydrate content was not significantly affected by either temperature or pCO_2_ level (*p* > 0.05, Figure 3B and Table 3). The cellular protein contents of both *Thalassiosira* sp. and *N. closterium f.minutissima* significantly decreased with rising pCO_2_ under 15°C (by 18.03 and 13.07%, respectively, Figures 3C,D). However, this was not the case for higher temperature. Warming also significantly decreased the cellular protein content of *Thalassiosira* sp. under 400 ppm pCO_2_, but not at higher pCO_2_ level, resulting in the highest value observed under ambient treatment (Figure 3C). The cellular protein content of *N. closterium f.minutissima* significantly decreased with an elevated temperature at both 400 and 1,000 ppm pCO_2_ (*p* < 0.05, Figure 3D).

**Table 3:** Interactive effects of CO_2_ and temperature on the physiological parameters of *Thalassiosira* sp. and *N. closterium f.minutissima*.

**Parameter**	***Thalassiosira*** **sp**.				***N. closterium f.minutissima***			
	**Two-way ANOVA**			**Type of interaction**	**Two-way ANOVA**			**Type of interaction**
	**CO_**2**_**	**T**	**Interaction**		**CO_**2**_**	**T**	**Interaction**	
Growth rate	^*^	^*^	^*^	A	ns	^*^	ns	A
**POC production rate**	ns	ns	ns	A	^*^	^*^	^*^	S
Chl *a*	ns	^*^	ns	A	ns	ns	ns	A
POP	ns	^*^	ns	A	ns	^*^	ns	A
BSi	ns	^*^	ns	A	^*^	^*^	ns	S
POC	ns	^*^	ns	A	^*^	ns	ns	S
PON	ns	^*^	ns	A	^*^	ns	ns	S
C:N	ns	^*^	ns	A	ns	ns	ns	S
C:P	ns	ns	ns	A	^*^	^*^	ns	S
Si:C	ns	ns	ns	A	^*^	^*^	^*^	S
N:P	ns	^*^	ns	S	^*^	^*^	ns	S
Si:P	ns	ns	ns	S	^*^	ns	ns	S
Protein	^*^	^*^	^*^	A	^*^	^*^	ns	A
Carbohydrate	ns	^*^	^*^	A	ns	ns	ns	A
Cell Size	ns	ns	ns	A	ns	ns	ns	S
Sinking Rate	^*^	^*^	ns	S	ns	^*^	^*^	S

“^*^”Represents significance and “ns” represents non-significance at the p = 0.05 level using two-way ANOVA test. “S” represents synergistic effects and “A” represents antagonistic effects.

The cellular Chl *a* content of *Thalassiosira* sp. was mainly affected by changes in temperature (*p* < 0.05). Warming caused a decrease by 30.53% and 25.91% at 400 and 1,000 ppm, respectively (Figure 3E). Changing pCO_2_ alone had no significant effect on the cellular Chl *a* content (Figure 3E). For *N. closterium f.minutissima*, the cellular Chl *a* content was not significantly affected by temperature or pCO_2_ level (*p* > 0.05, Table 3).

### Sinking Rate

**Figure 4:**
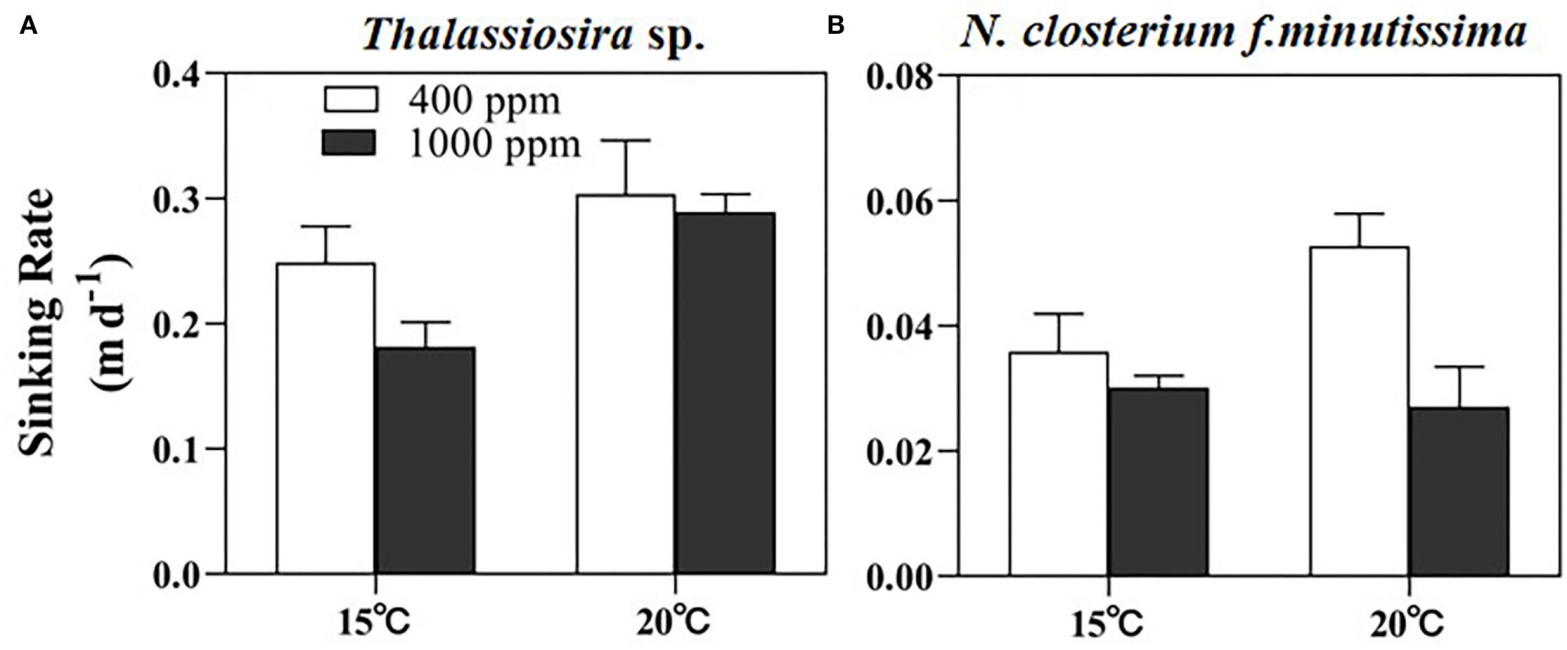
The sinking rate of *Thalassiosira* sp. **(A)** and *N. closterium f.minutissima* **(B)** under different pCO_2_ and temperature conditions. The error bars represent standard deviations of triplicate samples. The letters above the bars denote the statistically significant differences.

The sinking rate of *Thalassiosira* sp. (average of ~0.25 m day^−1^) was significantly higher than that of *N. closterium f.minutissima* (Figure 4). In general, the sinking rate of *Thalassiosira* sp. was lower at high pCO_2_, but higher at high temperature. The value was significantly decreased by 27.13% by OA at 15°C, and significantly increased by 22.00 and 59.42% by warming under 400 and 1,000 ppm pCO_2_, respectively (Figure 4A). OA significantly decreased sinking rate of *N. closterium f.minutissima* by 48.74% at 20°C, and warming significantly increased the sinking rate by 47.09% under 400 ppm pCO_2_, yielding the highest value (0.053 m day^−1^) in the 20°C, 400 ppm pCO_2_ treatment (*p* < 0.05, Figure 4B).

### Individual and Interactive Effects of CO_2_ and Temperature

The results of two-way ANOVA indicate that temperature had significant effects on most of the parameters of *Thalassiosira* sp., including the growth rate, cellular Chl *a*, POP, BSi, POC, PON and protein contents, and C:N and N:P ratios. CO_2_ only had significant effects on the growth rate and the protein content (Table 3). For the two-way factorial interaction, CO_2_ and temperature had significant antagonistic interactive effects on the growth rate and the cellular protein content of *Thalassiosira* sp. (Table 3 and Supplementary Table S2).

For *N. closterium f.minutissima*, temperature had significant effects on the growth rate, POC production rate, cellular POP and BSi contents, C:P, Si:C, and N:P ratios, and sinking rate. CO_2_ had significant effects on the POC production rate, cellular BSi, POC, and PON contents, C:P, Si:C, N:P, and Si:P ratios, and sinking rate (Table 3). In addition, CO_2_ and temperature had significant antagonistic interactive effects on growth rate and significant synergistic interactive effects on the POC production and Si:C ratio (Table 3 and Supplementary Table S2).

## Discussion

This study reveals that temperature played a more important role in regulating the physiology of both coastal diatom species, having significant effects on the growth, POC production, and elemental composition than rising pCO_2_. In addition, differential physiological responses between the two diatom species suggest potential species-specific effects of OA and warming and their interaction on the ecologically important phytoplankton group of marine diatoms.

### Differential Responses of Growth and POC Production

The OA had no significant effect on the growth rate of *N. closterium f.minutissima* in the present study, due to highly efficient CCMs processed by the cells, with saturated photosynthesis and growth under present-day CO_2_ condition (Reinfelder, 2011). However, rising pCO_2_ slightly promoted the growth of *Thalassiosira* sp., suggesting that the two species have different sensitivities under low temperature. The growth vs. pCO_2_ response curves (Supplementary Figure S1) of these species also showed a higher half saturation constant of pCO_2_ for *Thalassiosira* sp. than that for *N. closterium f.minutissima*. This is likely to be caused by the smaller surface area-to-volume ratio of the larger centric diatom cells, requiring higher CO_2_ level for the saturation of growth. Additionally, this is probably related to the cell morphology. Studies on both coastal and oceanic diatom communities reveal that CO_2_ enrichment favored the growth of centric diatoms more than pennate diatoms (Tortell et al., 2002; Feng et al., 2021).

Our results suggest that compared to rising pCO_2_, an elevated temperature played a more important role in promoting the growth rates of both diatom species. It has long been recognized that temperature is a very physical factor controlling the metabolic activities and growth of organisms (Talling, 1955). Warming generally stimulates the growth of phytoplankton cells, within thermal limits that vary according to species (Eppley, 1972; Goldman and Carpenter, 1974; Montagnes and Franklin, 2001). In our study, the 5°C warming significantly increased the growth rate of both species at pCO_2_ levels. Similarly, one previous study also showed that an increasing of temperature in 5°C from 15 to 20°C significantly increased the growth rate of *Thalassiosira weissflogii* by ~140% (Strzepek and Price, 2000). The maximum growth rates of eight diatom species showed a close to linear increase in response to temperature increases until their upper temperature thresholds were reached after 5 generations of acclimation (Montagnes and Franklin, 2001). In addition, at longer adaptation time scale, elevated temperature (25°C) increased the growth rate of diatom *T. weissflogii* (Zhong et al., 2021) over a selection period of ~380 days.

Compared to the large-celled centric diatom *Thalassiosira* sp., the growth of smaller *N. closterium f.minutissima* was promoted by warming at a larger extent in our study. Based on a previous study on the growth vs. temperature response norms of these two species (Supplementary Figure S1), the optimal temperature of the growth of *Thalassiosira* sp. (~19°C) was lower than that of *N. closterium f.minutissima* (~22°C). Warming also greatly promoted the POC production rate of *N. closterium f.minutissima*. Especially under higher pCO_2_ condition, the POC production rate was almost doubled, mainly due to increased growth rate and cellular POC content. However, this was not observed for *Thalassiosira* sp. It has been believed that phytoplankton species from warmer environment with higher thermal optima are generally smaller (Sal et al., 2015), and thus small-celled species may be favored more by the future warming trend. Toseland et al. (2013) reported that there was a larger mutational target for warming than for CO_2_ adaptation, which accelerated the speed of adaption because most enzymes were temperature-dependent. Furthermore, the mutations for the adaptation to warming may have, on average, a larger beneficial effect, in particular when the sample of mutational effects was larger (Orr, 2005). These results indicate that although OA and warming were both important global change related drivers, warming may be the dominant driver for the adaptive responses of diatoms in the future ocean.

### Biogeochemical Implications to Elemental Stoichiometry and Carbon Export

Phytoplankton elemental composition is one of the physiological properties that may influence the metabolic rates of phytoplankton, phytoplankton evolution, marine food web structure, the consequent carbon export to the deep ocean, and marine biogeochemistry (Finkel et al., 2009). Temperature changes mainly alter metabolic rates, and thus influence the diffusive rate of nutrients transported into cells and so may also affect the elemental composition of phytoplankton (Raven and Geider, 1988). Although the mechanisms for phytoplankton are still unclear (Yvon-Durocher et al., 2011), reducing body size has been proposed to be one of the universal responses of organisms to global warming (Daufresne et al., 2009). In general, every 1°C warming of temperature results in an average of 2.5% reduction in cell volume (Atkinson et al., 2003).

In our results, temperature greatly affected the elemental composition of the two diatom species. However, these two species have different widths of thermal tolerance (Supplementary Figure S1), thus having differential physiological responses to warming (Chen et al., 2021). For *Thalassiosira* sp., all the measured cellular elemental composition (POP, BSi, POC, and PON) decreased with warming, especially under 400 ppm pCO_2_ (Figure 3). Similarly, the cellular carbohydrate, protein, and Chl *a* contents of *Thalassiosira* sp. also decreased with an elevated temperature (Figure 4). Our results are consistent with previous research, which reported decreased cellular POP and BSi contents of centric diatom *Coscinodiscus* sp. with temperature rising from 16°C to 20°C (Qu et al., 2018). For *N. closterium f.minutissima*, the cellular POP, BSi, protein, and Chl *a* contents decreased at higher temperature. The decreased cellular elemental contents are likely resulted from decreased cell size with warming (Peter and Sommer, 2012, Table 2). However, the cellular POC and PON contents of *N. closterium f.minutissima* increased with warming under 1,000 ppm pCO_2_, probably caused by enhanced carbon uptake and protein synthesis when both pCO_2_ and temperature were elevated. Similarly, Tew et al. (2014) demonstrated that the cellular POC and PON of the two pennate diatoms (*Amphora coffeaeformis* and *Nitzschia ovalis*) tended to increase rising pCO_2_.

In addition, we observed significantly lower C:P and N:P ratios of *N. closterium f.minutissima* at 20°C compared to 15°C, implying an important effect of warming in shifting elemental stoichiometry (Hutchins and Boyd, 2016). This can be explained by the increased synthesis of phosphorus-rich ribosomes and associated rRNAs while decreased cellular protein synthesis at higher temperature (Toseland et al., 2013). Similarly, other research also reported lower C:P and N:P ratios in the diatoms *A. coffeaeformis* (Tew et al., 2014), *Chaetoceros wighamii* (Spilling et al., 2015), and *Pseudo-nitzschia subcurvata* (Boyd et al., 2016) along with warming.

Overall, OA had more significant effects on the elemental composition of the smaller pennate diatom *N. closterium f.minutissima*, compared to that of the large centric diatom *Thalassiosira* sp. (Table 3), indicating differential responses to OA among different diatom taxonomic groups (Wu et al., 2014). For *N. closterium f.minutissima*, the cellular POC and PON contents significantly increased with rising pCO_2_. Enhanced POC content under high pCO_2_ was an indicator of increased macromolecule synthesis. The cells tend to increase carbohydrate synthesis in response to increasing CO_2_ levels and convert it simultaneously to carbon-rich macromolecules (Biswas et al., 2017). In addition, both cellular BSi content and the Si:C ratio of *Thalassiosira* sp. and *N. closterium f.minutissima* decreased at higher temperature (20°C) and elevated CO_2_ levels, mainly because less incorporation of silicon into the diatom frustule occurred at higher growth rates (Martin-Jézéquel et al., 2000).

The species-specific responses of sinking rates to OA and warming may have implications for the carbon and silicon fluxes and thus the carbon export into the deep ocean (Armbrust, 2009). Our study showed that the sinking rate of larger-celled *Thalassiosira* sp. was almost 5-fold of that of the small-pennate diatom *N. closterium f.minutissima* (~0.05 m day^−1^, Figure 4). This agrees with the assumption of Stokes' law that the sinking rate is related to the square of the radius. One recent study also reported that even compared to centric diatoms with similar cell size, the sinking rate of pennate diatoms appears to be lower due to the different shapes between these two groups (Bienfang and Harrison, 1984). In addition, the sinking rate of diatoms may also be affected by the cellular BSi content, due to the ballasting effect of BSi, which facilitates the cells sinking into the deep layer. This explains the observed trend of slower sinking rate under high pCO_2_ condition for *Thalassiosira* sp. at 15°C and *N. closterium f.minutissima* at 20°C coincided with the decreased cellular BSi content caused by OA (Figures 2G,H). Similarly, a recent study on a natural phytoplankton community dominated by diatoms also observed a decreased sinking rate under OA, especially when temperature was elevated (Feng et al., 2021). Moreover, diatoms may reduce their density and, thereby, their sinking speed, by exchanging high for low molecular weight ions (Anderson and Siveene, 1978; Lavoie and Raven, 2020). This can be the cause of the observed increased sinking rate by warming. The production of transparent exopolymer particles (TEPs), which have lower density but facilitate the cells to form large aggregates, is another factor that can largely modulate the sinking rate of diatoms (Alldredge and Jackson, 1995).

It is noteworthy that the cultures used in this study were not axenic, potentially with bacteria coexisting in the culturing system. A variety of interactions occur between diatoms and specific groups of bacteria, including spanning mutualism, commensalism, and parasitism (Amin et al., 2012, 2015; van Tol et al., 2017). The TEPs released by diatom cells are often colonized and remineralized by bacteria (Passow, 2012). Although the bacteria abundance and production were not analyzed in our study due to some logistic constraints, a previous study reported reduced community respiration rate of bacteria associated with diatoms when temperature decreased from 15°C to 4°C (Iversen and Ploug, 2013). The higher respiration rate of bacteria in the warmer environment may result in more compact and denser packaging of the diatom particles after the disaggregation, and higher sinking rates (Iversen and Ploug, 2013). This probably explains the observed relatively higher sinking rate at 20°C in our study. If this trend can be extrapolated into the natural phytoplankton communities, the accelerated sinking of diatom aggregates may further affect the carbon export into the depth.

### Overall Individual Effects and Interactions Between OA and Warming

In conclusion, our results reveal differential sensitivities of different diatoms species to the changes in CO_2_/temperature regimes. Warming played more significant roles in regulating the physiology of both diatom species than OA. The future climate warming will lead to increased stratification and reduced nutrient concentrations, selecting for smaller phytoplankton cells, which dominate low-nutrient waters due to their low-nutrient requirements and high-nutrient uptake, resulting in global decreases in net primary and export production (Bopp et al., 2005; Finkel et al., 2009). However, the potential effect of elevated pCO_2_ has rarely been considered explicitly in biogeochemical models (Wu et al., 2014). When considering OA alone, OA may favor the growth and carbon fixation of larger-celled centric diatoms than the small-pennate diatom species. These results indicate the future increases in CO_2_ would favor increased growth rates of larger diatoms, especially in productive regions, which may act to increase the rate and efficiency of carbon export from the surface to the deep sea, and hence help to offset reduced export though warming. Furthermore, our study indicates that OA and warming had interactive effects on diatom physiology. The interaction between OA and warming showed either synergistic effects (on the POC production rate and Si:C of *N. closterium f.minutissima*) or mostly antagonistic effects (on the growth rate and elemental stoichiometry of *Thalassiosira* sp. and sinking rate of both species). This further emphasizes the importance of considering interactive/cumulative effects of multiple environmental drivers in the future coastal environment where various environmental conditions may change simultaneously (IPCC, 2021).

## Data Availability Statement

The original contributions presented in the study are included in the article/Supplementary Material, further inquiries can be directed to the corresponding author.

## Author Contributions

TC conducted the experiments, analyzed the data, and wrote the first draft of this manuscript. YF designed the study and was responsible for data analyses, made the contribution on results interpretation, and manuscript writing and revision. YW, TL, and JW were responsible for sample collecting and analyzing. WL and WZ contributed to the results discussion and manuscript revision. All authors contributed to the article and approved the submitted version.

## Funding

This work was financially supported by the Shanghai Frontiers Center of Polar Science (SCOPS), the National Natural Science Foundation of China grant (No. 41676160), the Tianjin Natural Science Foundation (19JCYBJC22900 and 19JCYBJC23600), and the Guangdong Natural Science Foundation (No. 2022A1515010656).

## Conflict of Interest

The authors declare that the research was conducted in the absence of any commercial or financial relationships that could be construed as a potential conflict of interest. The Reviewer ZZ declared a shared affiliation with the author WZ at the time of the review.

## Publisher's Note

All claims expressed in this article are solely those of the authors and do not necessarily represent those of their affiliated organizations, or those of the publisher, the editors and the reviewers. Any product that may be evaluated in this article, or claim that may be made by its manufacturer, is not guaranteed or endorsed by the publisher.
