# Supplementary material for: The Differential Responses of Coastal Diatoms to Ocean Acidification and Warming: A Comparison Between Thalassiosira sp. and Nitzschia closterium f.minutissima
Source: Front Microbiol. 2022 Jun 21;13:851149. doi: 10.3389/fmicb.2022.851149 (PMC9253669; doi:10.3389/fmicb.2022.851149)
Supplement: Supplementary file 1 [file Data_Sheet_1.docx]

Supplementary Material

**Supplementary Figure 1.** The growth rates of *Thalassiosira* sp. (a and c) and *N. closterium f.minutissima* (b and d) under different temperature and pCO_2_ conditions. Red line is the response curve of growth rate to temperature, fitted by $\text{f}(T)=\text{a}e^{bT}(1-(\frac{T-\text{z}}{w/2})^{2})$ in supplementary figure1(a and b), where f is a function of temperature T, w is the temperature range (also called temperature niche) when the growth rate is positive, and z is the growth rate Optimum temperature.). The red line is fitted by the equation f(X) = Vmax * (X / (X + Km)) in supplementary figure1(c and d), where Vmax is the maximum growth rate, Km is the half-saturation constant.

**Supplementary Figure 1.**


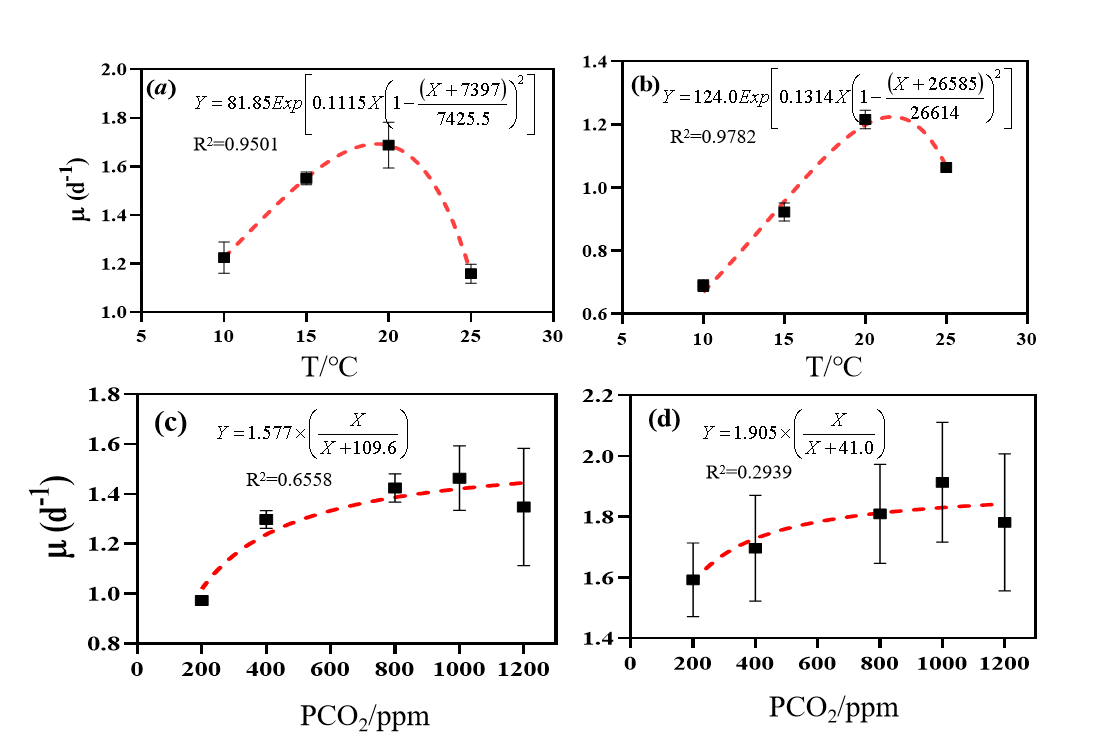


**Supplementary Table 1.** The seawater carbonate chemistry. pH and TA (total alkalinity) were directly measured values. TCO_2_, [HCO_3_^-^] and pCO_2_ were calculated using CO2sys.

| Treatment | pH | TA | TCO_2_ | [HCO_3_^-^] | Calculated pCO_2_ |
| --- | --- | --- | --- | --- | --- |
|  |  | μmol kg^-1^ | μmol kg^-1^ | μmol kg^-1^ | ppm |
| 15°C+400 ppm | 8.18 ± 0.009 | 2525.1 ± 13.08 | 2220.1 ± 4.08 | 2006.6 ± 6.43 | 429.8 ± 8.38 |
| 15°C +1000 ppm | 7.89 ± 0.02 | 2523.5 ± 22.98 | 2359.4 ± 10.30 | 2215.3 ± 14.29 | 916.4 ± 57.25 |
| 20°C +400 ppm | 8.23 ± 0.002 | 2509.5 ± 7.13 | 2187.11 ± 1.23 | 1954.21 ± 1.95 | 368.2 ± 2.06 |
| 20°C+1000 ppm | 7.94 ± 0.009 | 2511.1 ± 15.53 | 2339.7 ± 4.03 | 2187.7 ± 5.74 | 811.9 ± 19.62 |

**Supplementary Table 2.** The observed individual and multiplicative effects and calculated multiplicative effects of increased pCO_2_ (acidification) and warming on the physiological parameters of *Thalassiosira* sp. and *N. closterium f.minutissima*

| Parameter | *Thalassiosira* sp. | | | | | *Nitzschia closterium f.minutissima* | | | | |
| --- | --- | --- | --- | --- | --- | --- | --- | --- | --- | --- |
|  | Individual Effect (%) | | Multiplicative Effect (%) | | Type of Interaction | Individual Effect (%) | | Multiplicative Effect (%) | | Type of Interaction |
|  | OE_A_/% | OE_T_/% | OE_A+T_/% | ME_A+T_/% |  | OE_A_/% | OE_T_/% | OE_A+T_/% | ME_A+T_/% |  |
| Growth Rate | 13.61 | 26.38 | 9.88 | 43.58 | A | 3.73 | 34.08 | 28.99 | 39.08 | A |
| POC Production Rate | 10.79 | 2.96 | -4.47 | 14.07 | A | 54.52 | 24.77 | 198.5 | 92.79 | S |
| Chl *a* | -0.03 | -30.53 | -25.93 | -30.55 | A | -16.12 | -12.02 | -26.19 | -26.20 | A |
| POP | -18.28 | -30.05 | -30.86 | -42.83 | A | -26.55 | -27.97 | -35.42 | -47.10 | A |
| BSi | -17.06 | -29.68 | -33.23 | -41.67 | A | -33.28 | -19.57 | -55.61 | -46.34 | S |
| POC | -14.77 | -29.78 | -32.55 | -40.15 | A | 48.18 | -6.79 | 125.2 | 38.13 | S |
| PON | -5.30 | -41.97 | -34.52 | -45.05 | A | 86.59 | -7.34 | 133.1 | 72.89 | S |
| C:N | -7.53 | 21.12 | 3.33 | 12.01 | A | -1.89 | 3.90 | -15.39 | 1.94 | S |
| C:P | 4.88 | 0.67 | -2.35 | 5.58 | A | 134.3 | 25.77 | 212.2 | 194.7 | S |
| C:Si | 3.32 | 0.78 | 2.01 | 4.12 | A | 167.7 | 15.12 | 422.1 | 208.1 | S |
| N:P | 15.10 | -16.69 | -4.96 | -4.11 | S | 116.5 | 7.81 | 199.5 | 133.4 | S |
| Si:P | 1.51 | 0.16 | -4.12 | 1.68 | S | -10.90 | 8.62 | -32.10 | -3.22 | S |
| Protein | -16.94 | -18.03 | -22.70 | -31.92 | A | -16.03 | -23.89 | -31.53 | -36.09 | A |
| Carbohydrate | -3.49 | -43.34 | 5.60 | -45.32 | A | 18.30 | 8.10 | 7.67 | 27.88 | A |
| Cell Size | 0.003 | -2.62 | 0.22 | -2.61 | A | -3.33 | -1.51 | -7.50 | -4.79 | S |
| Sinking Rate | -27.13 | 22.00 | 16.18 | -11.10 | S | -15.60 | 47.09 | -24.60 | 23.56 | S |

“S” represents synergistic effects and “A” represents antagonistic effects.
